# Protective effect of *Phellinus linteus* polysaccharide extracts against thioacetamide-induced liver fibrosis in rats: a proteomics analysis

**DOI:** 10.1186/1749-8546-7-23

**Published:** 2012-10-18

**Authors:** Hualin Wang, Guang Wu, Hyoung Jin Park, Ping Ping Jiang, Wai-Hung Sit, Leo JLD van Griensven, Jennifer Man-Fan Wan

**Affiliations:** 1 Food and Nutrition Division, School of Biological Sciences, The University of Hong Kong, Hong Kong, SAR, China; 2 Department of Physiology, College of Medicine, Hallym University, 39 Hallymdaehak-gil Chuncheon, Gangwon-do, 200-702, South Korea; 3 Plant Research International, Department of Bioscience, Wageningen University, Wageningen, The Netherlands

## Abstract

**Background:**

The hepatoprotective potential of *Phellinus linteus* polysaccharide (PLP) extracts has been described. However, the molecular mechanism of PLP for the inhibition of liver fibrosis is unclear. This study aims to investigate the molecular protein signatures involved in the hepatoprotective mechanisms of PLP *via* a proteomics approach using a thioacetamide (TAA)-induced liver fibrosis rat model.

**Methods:**

Male Sprague–Dawley rats were divided into three groups of six as follows: Normal group; TAA group, in which rats received TAA only; and PLP group, in which rats received PLP and TAA. Liver fibrosis was induced in the rats by repeated intraperitoneal injections of TAA at a dose of 200 mg/kg body weight twice a week for 4 weeks. PLP was given orally at a dose of 50 mg/kg body weight twice a day from the beginning of the TAA treatment until the end of the experiment. The development of liver cirrhosis was verified by histological examination. Liver proteomes were established by two-dimensional gel electrophoresis. Proteins with significantly altered expression levels were identified by matrix-assisted laser desorption/ionization-time of flight/time of flight mass spectrometry and the differentially expressed proteins were validated by immunohistochemical staining and reverse transcription polymerase chain reaction.

**Results:**

Histological staining showed a remarkable reduction in liver fibrosis in the rats with PLP treatment. A total of 13 differentially expressed proteins including actin, tubulin alpha-1C chain, preprohaptoglobin, hemopexin, galectin-5, glutathione S-transferase alpha-4 (GSTA4), branched chain keto acid dehydrogenase hterotetrameric E1 subunit alpha (BCKDHA), glutathione S-transferase mu (GSTmu); glyceraldehyde-3-phosphate dehydrogenase (GAPDH); thiosulfate sulfurtransferase (TFT); betaine-homocysteine S-methyltransferase 1 (BHMT1); quinoid dihydropteridine reductase (QDPR); ribonuclease UK114 were observed between the TAA and PLP groups. These proteins are involved in oxidative stress, heme and iron metabolism, cysteine metabolism, and branched-chain amino acid catabolism.

**Conclusion:**

The proteomics data indicate that *P. linteus* may be protective against TAA-induced liver fibrosis via regulation of oxidative stress pathways, heat shock pathways, and metabolic pathways for amino acids and nucleic acids.

## Background

Most chronic liver diseases, including viral hepatitis (hepatitis B virus and hepatitis C virus), alcoholic liver disease, and biliary diseases [1], ultimately lead to liver fibrosis. Without effective treatments at an early stage, reversible liver fibrosis will lead to irreversible cirrhosis [2]. Oxidative stress may cause liver damage [3,4], and reducing oxidative stress by supplementation with antioxidants is effective for preventing liver fibrogenesis [5]. However, evidence for the efficacy of antioxidants, such as vitamin E and superoxide dismutase, in the treatment of human liver fibrosis has not been established [6].

*Phellinus linteus* (Berk. et Curt.) *Teng*, an orange-colored mushroom, belongs to the Hymenochaetaceae Basidiomycetes and has been considered useful in preventing and treating liver fibrosis and liver cancers owing to its strong anti-inflammatory, antioxidative, antiangiogenic, and anticancer properties [7-10]. *P. linteus* has been used in Chinese medicine for the treatment of tumors, menstrual irregularities, and liver-related illnesses [11]. Several reports from Korea and Japan have demonstrated that intake of *P. linteus* for a long time may induce spontaneous regression of hepatocellular carcinoma in patients with multiple metastases [12,13]. Some *in vivo* and *in vitro* studies have also demonstrated that *P. linteus* exerts antitumor effects on hepatocellular carcinoma [14-16].

Over the last decade, accumulating evidence suggests that *P. linteus* may protect the liver against fibrosis via its antioxidative property. A study in 2002 demonstrated that an extract of *P. linteus* was able to suppress carbon tetrachloride-induced late liver fibrosis by reducing peroxidation products, restoring the activities of catalase and superoxide dismutase, and reviving the expression of aerobic respiration enzymes [11]. Shon *et al.*[11] demonstrated that a *P. linteus* polysaccharide (PLP) fraction was able to inhibit cytochrome P450 isozymes in the liver. Furthermore, a retinoic acid derivative isolated from *P. linteus* was reported to decrease transforming growth factor-beta-induced early liver fibrosis by downregulating reactive oxygen species generation and suppressing the expression of several proteins [11].

Although antioxidation is an important mechanism by which *P. linteus* suppresses liver fibrosis, the molecular mechanism of the antioxidative effect of *P. linteus* is still unclear. To date, studies on *P. linteus*-mediated protection of the liver against injury have only found a few target molecules [17]. With the development of proteomics technology, it is possible to cover the expression of more proteins acting within a biological context to investigate the cellular processes involved in disease pathogenesis with high-throughput and in a quantitative manner [18,19].

In the present study, we aim to assess the hepatoprotective effects of *P. linteus* against thioacetamide (TAA)-induced liver fibrosis by high-resolution two-dimensional polyacrylamide gel electrophoresis (2-DE) coupled with mass spectrometry technology.

## Methods

### Preparation of PLP

Sang Hwang 125 capsules containing a lyophilized hot water extract of wild-type *P. linteus* were donated by Dr. Frankie Chan (Amazing Grace Health Products Limited Partnership, Thailand). Each Sang Hwang capsule contained 400 mg of pure extracts from natural *P. linteus*. The polysaccharides and glucan contents of the *P. linteus* natural compound are 53–63% and 24%, respectively, as previously reported by us [20]. This natural compound has been shown to possess strong antioxidative and immunomodulatory properties [21]. PLP was prepared by dissolving 100 g of freeze-dried powder from Sang Hwang 125 capsules in 1 L of distilled water, followed by the addition of 2 L of ethanol (Merck, Germany) at −20°C. The precipitated polysaccharides were collected by centrifugation at 3000 × g for 1 h, dissolved in a small volume of distilled water, and lyophilized. The resulting powder was stored at −20°C until use.

### Animal experiments

Eight-week-old male Sprague–Dawley rats (weighing approximately 200 g) were obtained from Hallym University (Korea). All rats were kept in an animal house under a 12-h/12-h light/dark cycle, with controlled temperature and humidity and free access to food and water. After 1 week of acclimatization, the rats were arbitrarily divided into three groups: Normal group; TAA group, in which rats received TAA only; and PLP group, in which rats received PLP and TAA. TAA (Sigma-Aldrich, USA) was intraperitoneally injected at a dose of 200 mg/kg body weight twice a week for 4 weeks. PLP was given orally at a dose of 50 mg/kg body weight twice a day from the beginning of the TAA treatment until the end of the experiment. All rats were euthanized after 4 weeks by intraperitoneal injection of 200 mg/kg sodium pentobarbital (Sigma-Aldrich, USA). Dissection was carried out, and liver samples were fixed in 10% buffered formalin solution (Surgipath, Germany) for histological staining. Tissues from the same portion of the liver were collected from the TAA and PLP groups for the proteomics analysis.

The study protocol was approved by the Hallym University, South Korea. Animal care complied with institutional guidelines.

### Histological examination of the liver

The fixed liver tissues were embedded in paraffin and sectioned at 5-μm thickness. For each liver sample, the stage of hepatic fibrosis was established. The liver sections were stained with Masson’s trichrome (Sigma-Aldrich, USA) and observed under NIKON model SE microscope (NIKON, Japan) to evaluate the degree of fibrosis.

### Sample preparation for proteomics analysis

Liver samples were snap-frozen in liquid nitrogen and stored at −80°C for the proteomics analysis. The frozen liver tissue samples from the TAA and PLP groups were disrupted with a tissue teaser (Biospec Products, USA) in a lysis buffer containing 25 mM HEPES, pH 7.5, 150 mM NaCl, 1 mM EDTA disodium salt, 1 mM dithiothreitol (DTT) (USB, USA), 1% (v/v) Triton X-100 (USB, USA), and 1% (v/v) Protease Inhibitor Cocktail Set III (Bio-Rad, USA). The superfluous salt in the extract was removed by incubation with 20% (w/v) trichloroacetic acid (TCA)-acetone solution and 20 mM DTT in acetone (Merck, Germany) for 4 hours at −40°C. The protein pellet was obtained by centrifugation at 15,800 × *g* for 30 min at 4°C. Excess TCA was removed by three washes with acetone containing 20 mM DTT. After air-drying, the protein pellet was resuspended in buffer comprising 7 M urea, 2 M thiourea, 100 mM DTT, 5% (v/v) glycerol, and 4% (w/v) 3-[(3-cholamidopropyl)dimethylammonio]-1-propanesulfonate (CHAPS) (USB, USA), and the resulting protein solution was stored at −80°C until 2-DE analysis. The protein concentration was determined by the Bradford assay (Bio-Rad, USA).

### Two-dimensional gel electrophoresis

The 2-DE procedures were performed according to our previous study [22] with some modifications. The tissue samples were processed in duplicate and a total of 12 gels (six for the TAA group and six for the PLP group) were used. For the first-dimension electrophoresis, 100-μg protein samples were mixed with 350 μL of rehydration buffer comprising 9.5 M urea, 2% (w/v) CHAPS, 0.28% (w/v) DTT, 0.002% (w/v) bromophenol blue (USB, USA) and 1% (v/v) immobilized pH gradient buffer (pH 3–10) (Bio-Rad, USA), and then applied to an Ettan IPGphor 3 isoelectric focusing electrophoresis system (GE healthcare, USA). The samples were rehydrated for 7 h before isoelectric focusing with the following programs: (a) linear increase up to 500 V over 1 h; (b) holding at 500 V for 2 h; (c) linear increase up to 10,000 V over 4 h; (d) linear increase up to 10,000 V over 3 h; and (e) final hold at 10,000 V to reach a total of 120,000 V × h. The focused immobilized pH gradient gel strips were equilibrated for 15 min in a solution comprising 50 mM Tris–HCl, pH 8.8, 6 M urea, 30% (v/v) glycerol, 2% (w/v) sodium dodecyl sulfate (SDS) and 20 mM DTT, followed by incubation with the same buffer containing 20 mM iodoacetamide (Sigma-Aldrich, USA) for another 15 min. The second-dimension separation was performed by 12.5% SDS polyacrylamide gel electrophoresis (PAGE) at a constant current of 30 mA for 30 min, followed by a 60-mA current for the rest of the analysis until the bromophenol blue line reach the bottom of the gels.

### Image acquisition and analysis

After the 2-DE, the gels were stained with SYPRO® Ruby Protein Stain (Bio-Rad, USA) according to the manufacturer’s protocol. The stained gels were scanned with a Molecular Imager PharosFX Plus System (Bio-Rad, USA) and analyzed by PDQuest 8.0 software (Bio-Rad, USA). Each expression level was calculated as the percentage volume (% vol), and exported for statistical analysis. The relative intensities of spots were used for comparison between the two groups, and only those spots with significant differences (≥ 1.5-fold increase or decrease; *P* < 0.05) were selected for protein identification.

### Protein identification

Spots showing differential expression (*P* < 0.05) between the TAA and PLP groups were sent to the Genome Research Centre (The University of Hong Kong, Hong Kong) for protein identification. The proteins were digested with sequencing grade modified trypsin (Promega, USA) and applied to matrix-assisted laser desorption/ionization-time-of-flight/time-of-flight (MALDI-TOF/TOF) mass spectrometer analysis using a 4800 MALDI TOF/TOF Analyzer (Applied Biosystems, USA). Matches between the experimental data and mass values calculated from a candidate protein were carried out by Mascot search engine (Matrix Science, UK) that uses MS data to identify proteins from the NCBInr database with taxonomy limited to *Rattus norvegicus*. The database allowed up to one missed cleavage and the mass tolerance was set as 75 ppm peptide limited by fixed modification of carbamidomethyl and variable modification of oxidation, with monoisotopic values. Mascot reported the molecular weight search (MOWSE) score, which is calculated by −10 × log10 (P), where P is the probability that the observed match is a random event. The P value is limited by the size of the sequence database being searched (limited by taxonomy), the conditions, and the settings of trypsin digestion. Each calculated value that falls within a given mass tolerance of an experimental value counts as a match. The accepted threshold is that an event is significant if it would be expected to occur at random with a frequency of < 5%. In this study, a protein match with a score of > 71 was regarded as significant.

### Western blot analysis for validation of differentially expressed proteins

Western blot analysis was employed to validate the proteomic data. Liver protein extracts were mixed with sample buffer (62.5 mM Tris–HCl, pH 6.8, 25% (v/v) glycerol, 2% (w/v) SDS, 350 mM DTT, and 0.01% (w/v) bromophenol blue) at a ratio of 1:1 and incubated in boiling water for 5 min. Aliquots of the samples (30 mg of protein) were separated by electrophoresis in 12.5% SDS-PAGE gels at constant voltage (120 V) and then transferred to polyvinylidene difluoride membranes (GE Healthcare, USA) using a TE77 PWR Semi-dry Transfer Unit (GE Healthcare, USA). The membranes were blocked with 5% (w/v) non-fat dry milk in phosphate buffer saline overnight at 4°C. The membrane was incubated with primary antibodies: anti-haptoglobin (1:1000), anti-hemopexin (1:1000;), anti-hemoglobin (1:1000), anti-GSTA4 (1:500), and anti-GSTmu (1:1000) (Abcam, USA) for one hour and then incubated with their corresponding secondary horseradish peroxidase-conjugated antibodies (Bio-Rad, USA) for another one hour. The blots were washed five times with 0.05% Tween-20 in phosphate buffer saline between steps. Proteins were detected with an enhanced chemiluminescence system (GE Healthcare, USA) and the band intensity was measured with the Quantity One software (Bio-Rad, USA).

### Reverse transcription polymerase chain reaction

Reverse transcription polymerase chain reaction (RT-PCR) was employed to verify the differentially expressed proteins identified by the proteomics analysis. Total RNA was extracted from liver samples in the TAA and PLP groups using TRIzol® (Invitrogen, USA). Aliquots of the total RNA (5 μg) were reverse-transcribed with Super Script III (Invitrogen, USA) in the presence of oligodeoxythymidylic acid primers (Sigma-Aldrich, USA) according to the manufacturer’s instructions. PCR was performed with an iCycler Thermal Cycler (Bio-Rad, USA). cDNA (0.5 μL) were used for each PCR amplification in a total reaction volume of 15 μL using iQ SYBR Green Super Mix (Bio-Rad, USA), and all reactions were performed in duplicate. A total of 11 genes were examined, including ribonuclease UK114, hemopexin, preprohaptoglobin, glutathione S-transferase alpha-4 (*Gsta4*), branched chain keto acid dehydrogenase heterotetrameric E1 subunit alpha (*Bckdha*), glyceraldehyde-3-phosphate dehydrogenase (*Gapdh*), haptoglobin, thiosulfate sulfurtransferase (*Tft*), betaine-homocysteine S-methyltransferase 1 (*Bhmt1*), quinoid dihydropteridine reductase (*Qdpr*), and dihydrofolate reductase (*Dhfr*) because they showed significantly different expression levels in the 2-DE proteomics analysis, plus ubiquitin C as an internal control. The primers in Table 1 were used for the PCR with mentioned annealing temperature. The amplification was initiated by 4 min denaturation at 94°C for 1 cycle, followed by 30 cycles at 94°C for 30 s, specially annealing temperature of each gene for 30 s, and 72°C for 1 min using a Bio-Rad Icycler PCR thermocycler 96 well thermal thermo cycler (Bio-Rad, USA). After the last cycle of amplification, samples were incubated for 7 min at 72°C. The PCR products were examined in 1% agarose gels stained with 0.01% SYBR® Safe DNA Gel Stain (Invitrogen, USA) and analyzed using Quantity One software (Bio-Rad, USA).

**Table 1:** Primers used for the PCR analyses

**Gene**	**Primer sequence (5’-3’)**	**Fragment size (bp)**	**Annealing temperature (°C)**
*Preprohaptoglobin*	F^1^: TGCCTATCTGCCTGCCTTC	316	58
	R^2^: GTGTCCTCCTCCGTGTCAT		
*Hemopexin*	F: AAGCCAGACTCAGATGTAA	479	55
	R: AAGCAGTAGTAGCGTTCA		
*Gsta4*	F: GGACCTGATGATGATGATTATC	446	54
	R: TATCTTGCCTCTGGAATGC		
*Bckdha*	F: AGCGTCACTTCGTCACCATT	547	60
	R: GCCTTCTCCTGTTCCTCATCC		
*Bhmt*	F: CAGACACCTTCCTACCTCAG	281	52
	R: CAGTTCACACCGACAATGG		
*Dhfr*	F: CTTGACGGCACTCTAAGC	304	52
	R: CTCCTTGTGGTGGTTCCT		
*Qdpr*	F: GATGTGGTGGAGAATGAAGAGG	241	56
	R: AGTGGCTAGAGATGGTGGATG		
*Gapdh*	F: CATGACCACAGTCCATGCCATC	451	60
	R: CACCCTGTTGCTGTAGCCATATTC		
*Uk114*	F: GCATGTCGTCAATAATCAGA	443	54
	R: CTCCAGAGTCAGCATCAG		
*Tft*	F: GGTTCATCAGGTGCTCTATCG	311	58
	R: CCAGGTCGTCTCCATCGTATA		
*Ubiquitin C*	F: TGGAGGTCGAGCCCAGTGTTA	105	58
	R: CCCAAGAACAAGCACAAGAAGGGCT		

^1^F: Forword.

^2^R: Reverse.

### Statistical analysis

All data are presented as the mean ± standard deviation (SD). The significance of differences in data between the groups was determined by one-way analysis of variance followed by the Tukey test for equality of variances using SPSS 17.0 (IBM, USA). Differences were considered statistically significant at *P* < 0.05.

## Results

### Histological assessment of liver fibrosis

**Figure 1:**
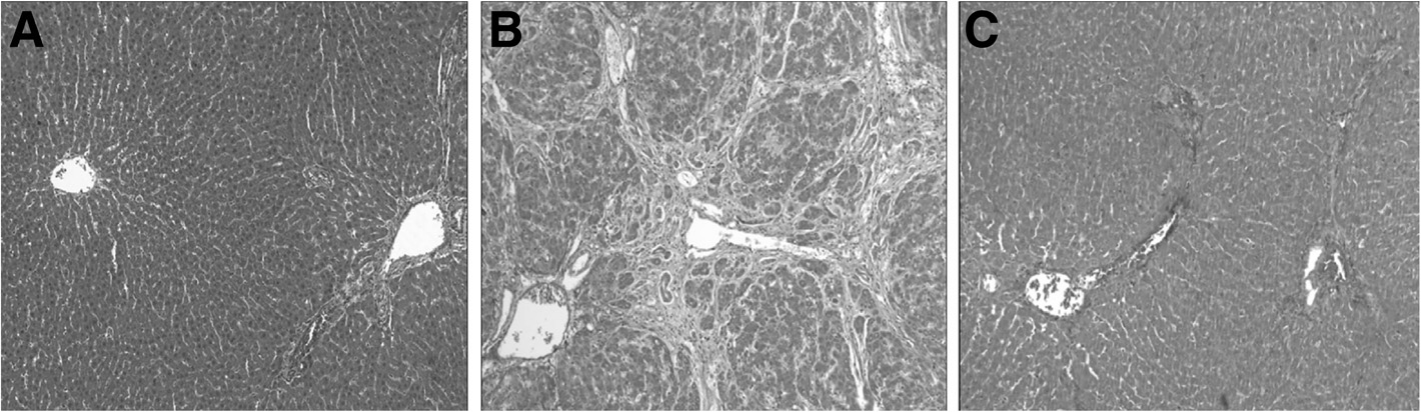
**Photomicrographs of rat livers.** The livers were sectioned at 5-μm thickness and the sections were stained with Masson’s trichrome. **A**: Normal group. **B**: TAA group. **C**: PLP group. Extended collagen deposition and large septa of the hepatic lobules are observed in the TAA-treated liver (**B**) compared with the normal liver (**A**). In addition, lymphoid infiltration is observed around the central and portal veins in the TAA-treated liver. PLP treatment markedly reduces the severity of the fibrosis and inflammation induced by TAA (**C**).

TAA treatment of rats for 4 weeks resulted in liver fibrosis, which was characterized by alterations in the quality of the hepatic extracellular matrix (Figure 1B&C), compared with the livers of rats in the Normal group (Figure 1A). Extended collagen deposition and large septa of the hepatic lobules were observed after 4 weeks of TAA treatment (Figure 1B). In addition, lymphoid infiltration was observed around the central and portal veins in the TAA-treated livers. PLP treatment markedly reduced the severity of the fibrosis and inflammation induced by TAA (Figure 1C).

### Identification of protein spots on 2-DE gels

**Figure 2:**
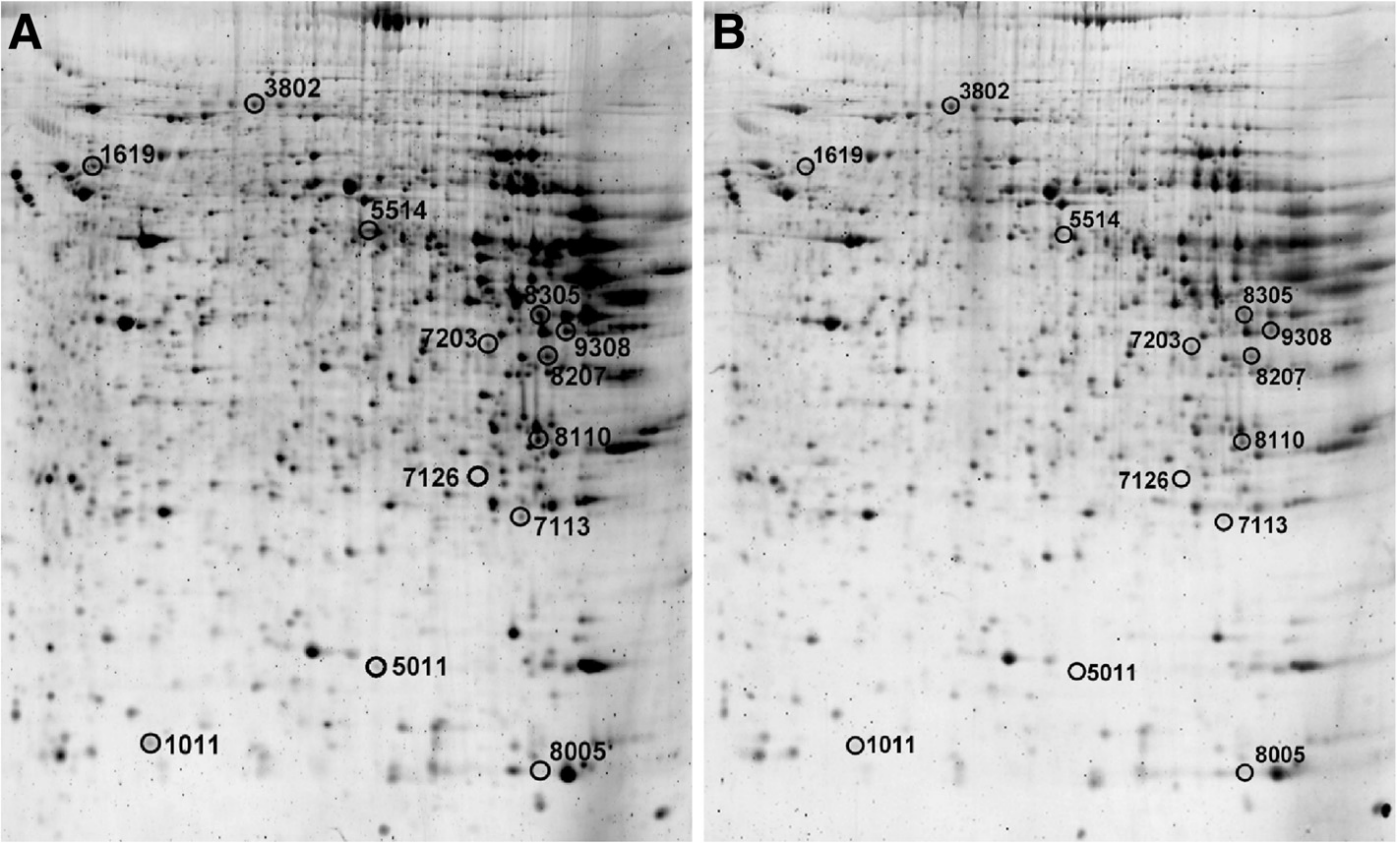
Representative 2-DE gel maps of the liver proteomes of rats in the TAA (A) and PLP (B) groups.

On each 2-DE gel, nearly 1000 individual protein spots were detected, and 13 spots with notable changes found by the PDQuest software between the PLP and TAA groups were identified by MS (Figure 2, Table 2). The proteins with increased expression levels in the PLP group compared with the TAA group included actin cytoplasmic 2, tubulin alpha-1C chain, galectin-5, BCKDHA, DHFR, preprohaptoglobin, GSTA4, QDPR, GAPDH, and TFT. The proteins with decreased expression levels in the PLP group compared with the TAA group were hemopexin, ribonuclease UK114, and BHMT1.

**Table 2:** Differentially expressed liver proteins between the TAA-induced liver fibrosis rats in the PLP and TAA groups

**Spot No.**^**1**^	**Protein name**	**GenInfo identifier**^**2**^	**Protein score**^**3**^	**Expression quantity (×10**^**2**^**) TAA**	**Expression quantity (×10**^**2**^**) PLP**	**Expression change (PLP/TAA)**	***P***	***pI***^**4**^	***Mr*(kDa)**^**4**^
1011	actin, cytoplasmic 2	gi|4501887	65	62.6 ± 24.5	97.1 ± 13.0	1.6	0.012	5.31	42.1
1619	tubulin alpha-1C chain	gi|58865558	254	29.4 ± 13.0	55.4 ± 14.0	1.9	0.014	4.96	50.6
3802	hemopexin	gi|122065203	262	100.5 ± 34.1	61.4 ± 15.4	−1.6	0.028	7.58	52.0
5011	galectin-5	gi|785053	120	11.5 ± 9.5	75.3 ± 44.9	6.5	0.028	6.95	15.5
5514	Bckdha protein	gi|59808237	88	41.3 ± 9.1	61.0 ± 11.5	1.5	0.008	6.4	37.6
7113	dihydrofolate reductase	gi|18426814	100	49.3 ± 14.7	73.8 ± 14.0	1.5	0.015	6.77	21.7
7126	Glutathione S-transferase alpha-4	gi|157820217	67	15.1 ± 7.6	28.9 ± 11.3	1.9	0.033	6.77	25.6
7203	preprohaptoglobin	gi|204657	75	54.8 ± 20.6	92.5 ± 16.2	1.7	0.005	7.16	30.4
8005	ribonuclease UK114	gi|47168636	159	172.2 ± 69.2	78.9 ± 45.1	−2.2	0.020	7.79	14.4
8110	quinoid dihydropteridine reductase, isoform CRA_c	gi|149047263	135	83.7 ± 22.3	128.5 ± 25.5	1.5	0.009	9.69	27.9
8207	betaine-homocysteine S-methyltransferase 1	gi|13540663	142	128.6 ± 42.6	65.1 ± 12	−2.0	0.006	8.02	45.4
8305	glyceraldehyde-3-phosphate dehydrogenase	gi|8393418	130	141.6 ± 69.1	225.8 ± 44.3	1.6	0.035	8.14	36.1
9308	thiosulfate sulfurtransferase	gi|57528682	256	63.8 ± 15.7	99.7 ± 22.8	1.6	0.010	7.71	33.6

^1^Spot no.: automatically assigned by the PDQuest software.

^2^GenInfo identifier: sequence identification number assigned by GenBank.

^3^Protein score: generated by the MS identification system.

^4^*Mr* and *pI*: relative molecular mass (*Mr*) and isoelectric point (*pI*) generated by the MS system.

### Western blot analysis for validation of differentially expressed proteins in the proteomics analysis

**Figure 3:**
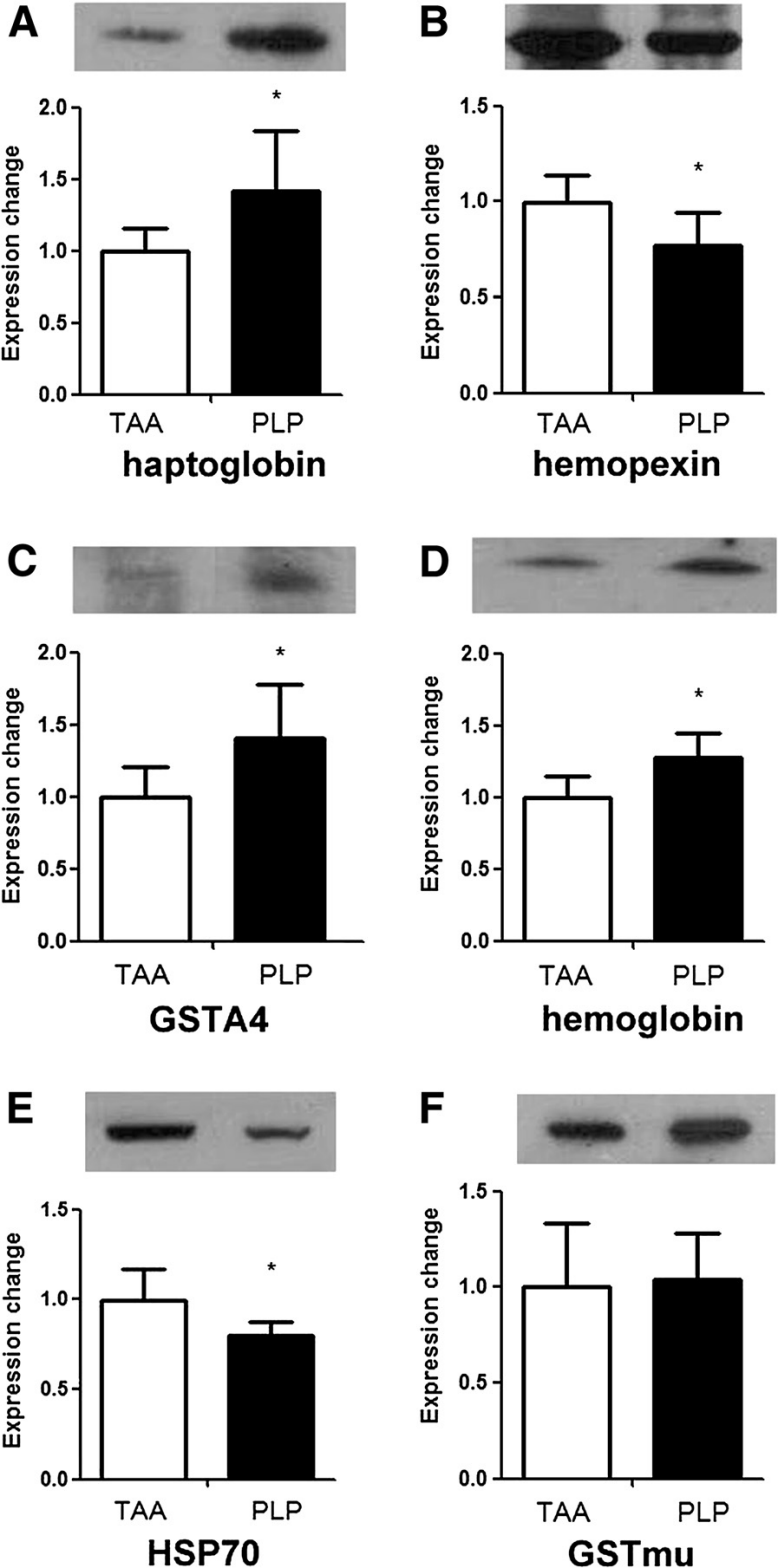
**Western blot measurements of the haptoglobin, hemopexin, GSTA4, hemoglobin, HSP70, and GSTmu expression levels in the TAA-induced fibrotic livers with and without PLP treatment.** The protein expression levels are presented as means ± SD (*N* = 6). *P < 0.05 *vs.* the TAA group.

Owning to the limitations of anti-rat protein antibodies, many of the identified differentially expressed proteins could not be measured by western blot analysis. Haptoglobin, hemopexin, heat-shock protein 70 (HSP70), and GSTA4 were successfully measured and used to validate the results obtained in the proteomic analysis. The western blot results were in general agreement with the differentially expressed proteins obtained in the proteomic analysis. As shown in Figure 3, the level of hemopexin (*P* = 0.049) was lower and the levels of haptoglobin (*P* = 0.042) and GSTA4 (*P* = 0.040) were much higher in the PLP group compared with the TAA group. The levels of hemoglobin (*P* = 0.047) and HSP70 (*P* = 0.041) were higher in the PLP group than in the TAA group. GSTmu did not show a significant difference in the western blot analysis.

### Quantitative RT-PCR for gene expression analysis

**Figure 4:**
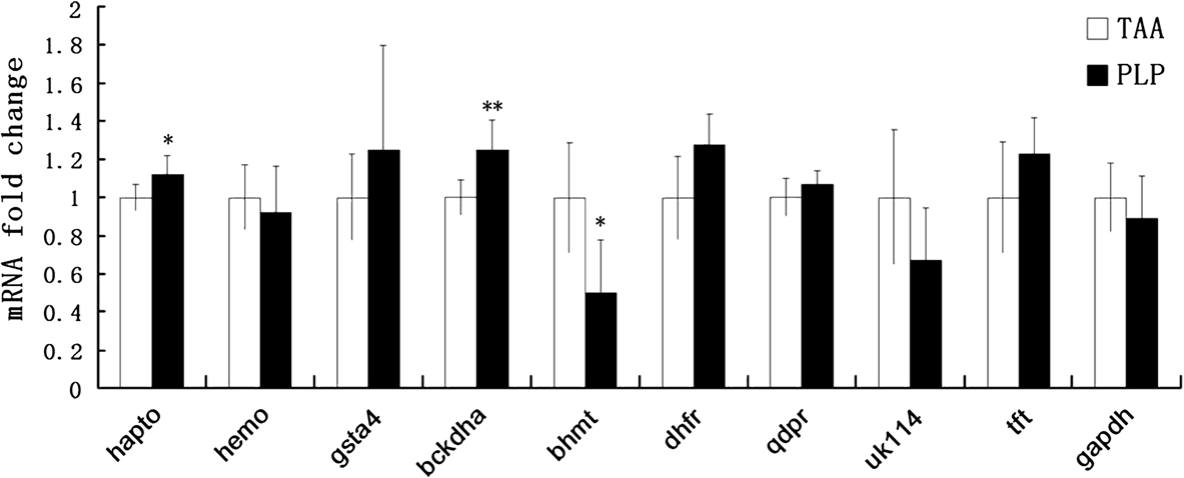
**Semiquantitative RT-PCR analyses of the effects of PLP on the gene expression changes of significantly altered proteins during TAA-induced liver fibrosis.** The mRNA expression levels are presented as means ± SD (*N* = 6). *P < 0.05,**P < 0.01 *vs.* the TAA group.

To investigate whether the expression changes of the identified proteins occurred at the transcriptional level, we determined the mRNA expression changes of these proteins by semiquantitative RT-PCR. As shown in Figure 4, the mRNA expression of many of the identified genes changed in a similar tendency as their protein expression change showed in 2D proteomic results, suggesting that the effects of *P. linteus* were exerted at the protein expression level, *i.e.* focused on the translation and post-translation steps. Haptoglobin, BCKDHA, and BHMT showed significant differences between the TAA and PLP groups.

## Discussion

The present study demonstrates that a natural product derived from *P. linteus* was able to protect against liver fibrosis induced in rats by chronic insult with TAA. The histopathological data clearly showed a reduction in collagen accumulation in the liver with PLP treatment. The present study thus supports the earlier findings that *P. linteus* possesses the capability to suppress liver injury [17,23] and exhibits strong and specific inhibitory activities to reduce peroxidation products and increase antioxidant enzymes in the liver [17,23].

**Figure 5:**
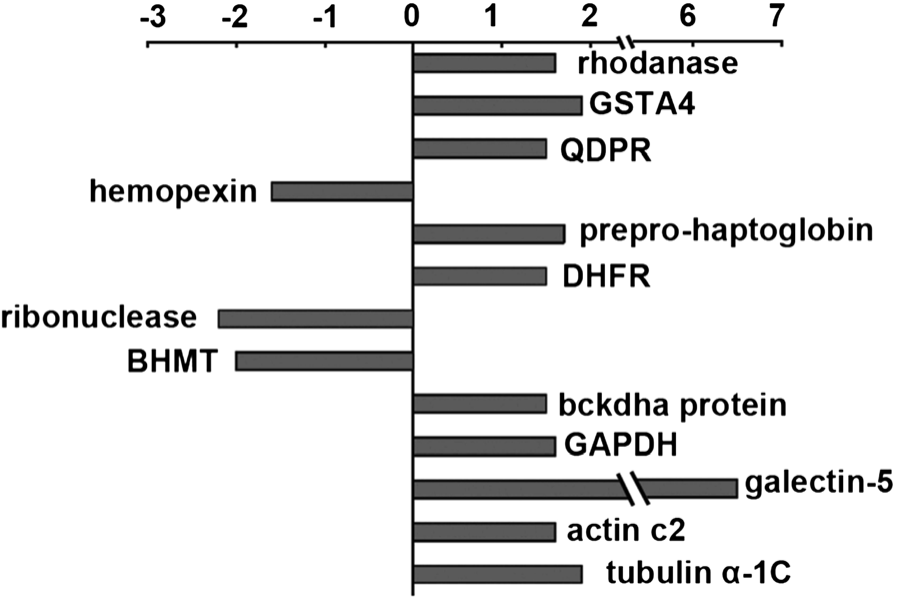
Effects of PLP on the expression changes of significantly altered proteins involved in TAA-induced liver fibrosis (PLP/TAA).

By using a 2-DE gel proteomics approach, we identified 13 differentially expressed hepatic proteins in the TAA-induced liver fibrosis rats in response to PLP treatment. Of these, 10 proteins showed increased expression and three proteins showed reduced expression, and the expression changes ranged from ±1.5-fold to ±2.5-fold (Figure 5). When these proteins are categorized according to their biochemical and physiological functions, we found associations with oxidative responses, molecular chaperones, heme and iron metabolism, cysteine metabolism, branched-chain amino acid metabolism, energy metabolism, and glutathione metabolites (Table 3). Among these 13 proteins, the regulation of hemopexin, preprohaptoglobin, GSTA4, BHMT, BCKDHA, QDPR, DHFR, and galectin-5 expression could be important in the protective effects of *P. linteus* against liver fibrosis.

**Table 3:** Major biofunctions of the identified proteins

**Protein name**	**Subcellular location**	**Major functions**
**Anti-oxidant effects**		
hemopexin	Extracellular region	The highest binding affinity for heme, iron metabolism
preprohaptoglobin	Extracellular region	The highest binding affinity for hemoglobin
glutathione S-transferase alpha-4 (GSTA4)	Cytoplasm	GSH-related detoxification
betaine-homocysteine S-methyltransferase 1 (BHMT1)	Cytoplasm	cysteine metabolism and GSH synthesis regulation
Bckdha protein	Mitochondrion matrix	Branched-chain amino acids catabolism
**Liver amelioration**		
dihydrofolate reductase (DHFR)	Cytoplasm	Synthesis of nucleic acid precursors
quinoid dihydropteridine reductase (QDPR)	Cytoplasm. Synaptosome	Tetrahydrobiopterin recycle, amino acid metabolism
glyceraldehyde-3-phosphate dehydrogenase (GAPDH)	Cytoplasm. Nucleus.	Glucose metabolism, initiation of apoptosis
ribonuclease UK114	Mitochondrion. Cytoplasm. Nucleus. Peroxisome	Translational inhibition
galectin-5	Cytoplasm Cell surface of rat reticulocytes and erythrocytes	Erythrocyte differentiation and reticulocyte maturation

The proteomic data showed that the expression of preprohaptoglobin was 1.7-fold higher while that of hemopexin was 1.6-fold lower in the PLP group compared with the TAA group. The expression changes of these two proteins were validated by western blot analysis (Figure 3). The increase in hemopexin and decrease in haptoglobin are potential markers for fibrosis because of their involvement in the regulation of liver iron homeostasis [24]. The aspect of whether the protective effect of PLP against the TAA-induced liver fibrosis occurred *via* the regulation of iron homeostasis cannot be concluded in the present study, because the liver and serum iron concentrations were not determined. In a previous study, chelation of ferrous ions by *P. linteus* was described, and PLP was able to protect hepatocytes against iron overload-mediated oxidative stress [21]. Iron homeostasis regulation has been suggested as a potential PLP treatment target in liver fibrosis [25].

Glutathione (GSH) plays an important role in cellular detoxification, because it effectively scavenges free radicals and other reactive oxygen species. In GSH-related antioxidative detoxification, glutathione S-transferases (GSTs) play central role; GSTA4 plays a role in the cellular defense against oxidative stress and lipid oxidation during liver injury [26]. Dwivedi *et al.*[27] demonstrated that mGSTA4 null (−/−) mice showed much quicker and greater carbon tetrachloride-induced hepatotoxicity than wild-type (+/+) mice. In the present study, the expression of GSTA4 was 1.9-fold higher in the PLP group than in the TAA group, and the change was confirmed by western blot analysis. The upregulated expression of GSTA4 might protect the liver against the injury and oxidative stress induced by TAA. However, the western blot analysis did not show a significant change in GSTmu between the PLP and TAA groups. These results could arise through non-specificity of the antibody for GSTmu or because GSTA4 was likely to be regulated by *P. linteus.*

The expression of BHMT was 2-fold lower in the PLP group than in the TAA group, suggesting that homocysteine was inclined to be converted to cysteine in the transsulfuration reaction, generating more cysteine for GSH synthesis. *P. linteus* may promote the accumulation of substrates for GSH synthesis, cysteine and glutamate [28], by regulating the expression levels of BHMT and BCKDHA [29]. BHMT reduces the conversion of homocysteine to cysteine by catalyzing the remethylation of homocysteine back to methionine [30].

Branched-chain amino acid (BCAA) catabolism is an important intercellular source of glutamate [31]. The branched-chain α-keto acid dehydrogenase (BCKD) complex is the rate-limiting enzyme for the whole BCAA catabolism. The *Bckdha* gene encodes the E1 α subunit of the BCKD [32]. The expression of BCKDHA was 1.5-fold higher in the PLP group than in the TAA group, suggesting that more glutamate was generated for GSH synthesis in the PLP group.

Several proteins that showed higher expression in the PLP group are involved in amino acid metabolism and nucleic acid metabolism. These include BCKDHA (1.5-fold), QDPR (1.6-fold), and DHFR (1.5-fold). In clinical treatment of liver diseases, supplementation with BCAAs is considered useful to relieve protein malnutrition [33,34]. QDPR is an enzyme that takes part in the tetrahydrobiopterin recycling pathway, and tetrahydrobiopterin is the precursor of phenylalanine and tyrosine [35]. The higher expression of QDPR in the PLP group suggests that PLP may expedite protein and nucleic acid synthesis in the fibrotic liver. DHFR is important for regulating the cellular amount of tetrahydrofolate, which is essential for purine and thymidylate synthesis [36,37]. The higher expression of DHFR in the PLP group indicates that PLP may aid in the regeneration of liver injury. The expression of ribonuclease UK114, a translational inhibitor mostly present in the liver and kidney, was 2.2-fold lower in the PLP group, meeting the requirement for protein synthesis for liver regeneration. In a clinical study, downregulation of ribonuclease UK114 was observed in human hepatocellular carcinoma [38].

GAPDH catalyzes a step of glycolysis. The expression of GAPDH was 1.6-fold higher in the PLP group, suggesting a higher energy requirement for liver amelioration. Several studies have illustrated that GAPDH may work in non-metabolic processes, such as transcription regulation [39] and apoptosis initiation [40,41]. This may be another reason for the upregulation of GAPDH in the PLP group.

Galectins comprise a family of evolutionarily conserved glycan-binding proteins that take part in acute and chronic inflammation [42,43]. Galectin-5 contributes to erythrocyte differentiation and reticulocyte maturation, but its function in liver injury remains unclear [44,45]. The much higher expression of galectin-5 in the PLP group suggests that PLP may promote erythropoiesis, inflammation regulation, and liver regeneration.

**Figure 6:**
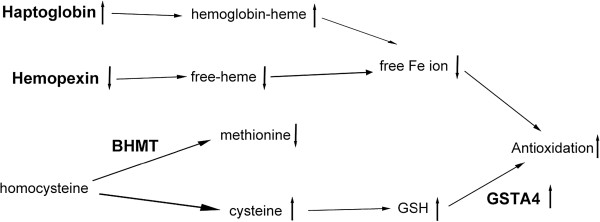
**Proposed mechanistic pathways for the protective effect of PLP against TAA-induced liver fibrosis in rats.** Liver proteins with significant expression changes detected by the 2-DE proteomics analysis are used to construct the possible pathways. These include the antioxidant system, iron metabolism regulation pathways (haptoglobin, hemopexin), and amino acid and nucleic acid metabolic pathways (homocysteine, BHMT, GSTA4). Upward arrowheads indicate upregulation or increasing, and downward arrowheads indicate downregulation or decreasing. BCAAs: branched-chain amino acids; BCKAs: branched-chain α-keto acids; BC acyl-CoA: branched-chain acyl-CoA.

Based on the proteomics data, we propose that the antioxidant pathway, iron metabolism pathway, and metabolic regulation of amino acids and nucleic acids are a few key networks involved in the hepatoprotective effect of PLP against TAA (Figure 6). Our western blot analyses further indicated that the PLP-mediated protection against TAA-induced hepatic injury involves the heat shock pathway. HSP70 has a crucial cytoprotective function mediated by its function as a molecular chaperone. A high level of HSP70 is a stress marker for liver injury [46,47]. The aspect of whether the reduced level of HSP70 represented a less inflammatory state of the TAA-treated liver with PLP treatment awaits confirmation by functional proteomics analyses in future studies.

## Conclusion

The present study has demonstrated that PLP can protect rats against TAA-induced liver fibrosis in at least two possible ways: 1) protection of the liver against oxidative stress, especially by scavenging of iron-related free radicals; and 2) regulation of the metabolism of amino acids and nucleic acids for liver amelioration. Our findings provide novel molecular mechanisms for the protective effects of *P. linteus* against liver fibrosis.

## Abbreviations

PLP: *Phellinus linteus* polysaccharide; TAA: Thioacetamide; 2-DE: Two-dimensional polyacrylamide gel electrophoresis; MALDI-TOF/TOF MS: Matrix-assisted laser desorption/ionization-time-of-flight/time-of-flight mass spectrometry; RT-PCR: Reverse transcription polymerase chain reaction; GSTA4: Glutathione S-transferase alpha-4; BCKDHA: Branched chain keto acid dehydrogenase heterotetrameric E1 subunit alpha; GSTmu: Glutathione S-transferase mu; GAPDH: Glyceraldehyde-3-phosphate dehydrogenase; TFT: Thiosulfate sulfurtransferase; BHMT1: Betaine-homocysteine S-methyltransferase 1; QDPR: Quinoid dihydropteridine reductase; DTT: Dithiothreitol; TCA: Trichloroacetic acid; CHAPS: 3-[(3-cholamidopropyl)dimethylammonio]-1-propanesulfonate; SDS: Sodium dodecyl sulfate; PAGE: Polyacrylamide gel electrophoresis; MOWSE: Molecular weight search; DHFR: Dihydrofolate reductase; HSP70: Heat shock protein 70; GSH: Glutathione; GSTs: Glutathione S-transferases; BCAA: Branched-chain amino acid; BCKD: Branched-chain α-keto acid dehydrogenase.

## Competing interests

The authors declare that they have no competing interests.

## Authors’ contributions

JMFW conceived and designed the study. HLW performed experiments, data acquisition, and results interpretation. HJP designed and GW conducted the animal experiments. PPJ and WHS performed the proteomics analysis. LJLDvG coordinated the study. JMFW, HLW, and LJLDvG wrote the manuscript. All authors read and approved the final version of the manuscript.
